# Twelve‐Month results from multicenter, open‐label, randomized controlled clinical trial comparing differential target multiplexed spinal cord stimulation and traditional spinal cord stimulation in subjects with chronic intractable back pain and leg pain

**DOI:** 10.1111/papr.13066

**Published:** 2021-08-27

**Authors:** Michael Fishman, Harold Cordner, Rafael Justiz, David Provenzano, Christopher Merrell, Binit Shah, Julian Naranjo, Philip Kim, Aaron Calodney, Jonathan Carlson, Richard Bundschu, Mahendra Sanapati, Vipul Mangal, Ricardo Vallejo

**Affiliations:** ^1^ Center for Interventional Pain and Spine Exton Pennsylvania USA; ^2^ Florida Pain Management Associates Sebastian Florida USA; ^3^ Oklahoma Pain Physicians Oklahoma City Oklahoma USA; ^4^ Pain Diagnostics and Interventional Care, LLC Sewickly Pennsylvania USA; ^5^ Coastal Carolina Research Center Charleston South Carolina USA; ^6^ Carolinas Research Institute PLLC Huntersville North Carolina USA; ^7^ South Florida Clinical Research South Miami Florida USA; ^8^ Precision Spine Care Tyler Texas USA; ^9^ Hawaii Pain and Spine Kailua Hawaii USA; ^10^ Coastal Orthopedics Bradenton Florida USA; ^11^ Global Scientific Innovations, LLC Evansville Illinois USA; ^12^ National Spine and Pain Center Oxon Hill Maryland USA; ^13^ National Spine and Pain Centers Bloomington Illinois USA

**Keywords:** back pain, differential target multiplexed, randomized controlled trial, spinal cord stimulation

## Abstract

**Background:**

Spinal cord stimulation (SCS) is a well‐established treatment for chronic intractable pain of the trunk and/or limbs; however, low back pain (LBP) is difficult to treat using traditional SCS. Differential Target Multiplexed spinal cord stimulation (DTM SCS) is an advanced approach inspired from animal studies demonstrating improved pain‐related behavior and pain‐relevant biological processes.

**Objective:**

The purpose of this study was to compare the effectiveness of DTM SCS and traditional SCS in treating chronic LBP and leg pain (LP).

**Methods:**

This prospective, postmarket randomized controlled trial compared DTM SCS to traditional SCS in patients with chronic LBP and LP. Primary end point was LBP responder rate (percentage of subjects with ≥ 50% relief) at 3 months. Noninferiority and superiority were assessed. Other outcomes included mean change in back and leg pain, responder rates, disability, global health, satisfaction, and safety profile throughout the 12‐month follow‐up.

**Results:**

One hundred twenty‐eight subjects were randomized across 12 centers (67 DTM SCS and 61 traditional SCS). Of the 94 patients implanted, 46 subjects in each group completed the 3‐month assessment. LBP responder rate of 80.1% with DTM SCS was superior to 51.2% with traditional SCS (*p* = 0.0010). Mean LBP reduction (5.36 cm) with DTM SCS was greater than reduction (3.37 cm) with traditional SCS (*p* < 0.0001). These results were sustained at 6 months and 12 months. Safety profiles were similar between treatment groups.

**Conclusion:**

Superiority of DTM SCS compared with traditional SCS for chronic LBP was demonstrated. Clinical improvements provided by DTM SCS were sustained over 12 months and are expected to significantly impact the management of chronic LBP.

**Key Points**

- A multicenter randomized controlled trial compared differential target multiplexed SCS (DTM SCS) and traditional SCS for the treatment of intractable chronic low back pain and leg pain
- Responder rate (% of subjects reporting at least 50% relief) for low back pain was superior with DTM SCS when compared to traditional SCS
- Additional benefits of DTM SCS were observed in improvements in quality of life, degree of disability, and subject satisfaction
- Benefits of DTM SCS for low back pain and leg pain were sustained through the 12‐Month followup
- DTM SCS and traditional SCS showed comparable acceptable safety profiles
- DTM SCS provided robust and positive benefits that were sustained over time

## INTRODUCTION

Chronic low back pain (LBP) is the most frequent pain condition afflicting at least one‐third of the American population, resulting in detrimental effects on quality of life and healthcare utilization, representing one of the top 3 causes of worldwide disability.^1^, ^2^, ^3^ Effective, long‐term pharmacological, interventional, and surgical treatments remain elusive.^4^ Spinal cord stimulation (SCS) is a well‐established treatment for chronic LBP and leg pain (LP).^5^, ^6^ Traditional SCS provides modest long‐term relief for patients with LBP.^7^, ^8^, ^9^, ^10^ Inadequate pain relief is the most common cause of SCS explantation.^11^, ^12^ Attempting to improve outcomes in this recalcitrant patient population, novel stimulation approaches have been introduced.^13^, ^14^, ^15^ Recently, SCS subject matter experts questioned the role of Aβ fiber activation in providing analgesia and developed innovative programming strategies. One such innovation is differential target multiplexed (DTM) SCS.

Vallejo et al.^16^ developed DTM SCS after demonstrating that conventional SCS modulated gene expression in the spinal cord at the site of stimulation, and the dorsal root ganglion corresponding with the nerve injured in an animal model of neuropathic pain. The DTM approach uses multiple electrical signals for modulating glial cells and neurons and rebalance their interactions.^17^, ^18^ Using animal models of neuropathic pain, the group demonstrated improved pain‐related behavior and modulation of pain‐relevant biological processes compared to conventional preclinical parameters. Preclinical work inspired a prospective, open‐label feasibility study in patients with LBP, in which conceptual learnings from preclinical studies were translated and further optimized for applications in humans. This short‐term trial phase study demonstrated that responder rate (percentage of patients with ≥ 50% LBP relief) was greater when patients were treated with DTM SCS than with traditional SCS.^19^ DTM SCS utilizes multiplexed electrical pulses that can be different from one another in aspects such as frequencies, pulse widths, charge balancing, and amplitudes. Settings for DTM SCS programs fall within approved labeling for the US Food and Drug Administration (FDA) and Continuing Education (CE) markings. Using a commercially available SCS system capable of delivering DTM SCS, we conducted a postmarket, open‐label, multicenter, prospective, randomized controlled trial (RCT) that evaluated the safety and efficacy of DTM SCS compared to traditional SCS in patients with LBP and LP. The noninferiority and superiority of DTM SCS versus traditional SCS for treating LBP was evaluated. Treatment outcomes of this RCT are presented here.

## METHODS

### Study design and patient selection

This multicenter, prospective, open‐label, postmarket RCT was designed to assess DTM SCS as compared with traditional SCS in subjects with intractable LBP and LP. The study was conducted at 12 investigational sites across the United States in compliance with the US Code of Federal Regulations, Good Clinical Practice Guidelines, and the 18th World Medical Assembly of Helsinki. The study protocol and informed consent forms were approved by the Western Institutional Review Board, Puyallup, WA. The study was registered with clinicaltrials.gov (NCT03606187). Key eligibility criteria are listed in Table 1.

**TABLE 1:** Key eligibility criteria

Inclusion	Exclusion
Adults (> 18 years old) Candidate for SCS system per labeled indication (back and leg pain) Average back pain intensity ≥ 5.0 cm on the 10.0 cm VAS with moderate to severe chronic leg pain at the time of enrollment Stable pain medication regime for at least 30 days prior to enrollment Willingness to not increase pain medications from baseline through the 3‐month visit	A medical, anatomic, and/or psychosocial condition that contraindicate the SCS neurostimulation system An existing, active implanted device Mechanical spine instability Experience within 30 days prior to enrollment of an interventional procedure and/or surgery to treat back and/or leg pain, which provided significant pain relief Unresolved major issues of secondary gain (e.g., social, financial, and legal)

Abbreviations: SCS, spinal cord stimulation; VAS, visual analog scale.

### Randomization and masking

Qualified subjects were randomized by a centralized electronic system after completing baseline assessments, using random permuted blocks with a 1:1 allocation ratio to either DTM SCS or traditional SCS. Practical considerations as outlined in the limitations section precluded masking of subjects and investigators.

### Procedures

Randomized subjects underwent a trial phase lasting up to 10 days. Investigators placed cylindrical percutaneous leads in the epidural space as described in the Lead Implant Manual.^20^ Leads were connected to an external neurostimulator, and stimulation therapy was programmed according to the allocated treatment. Those who had a “successful trial phase” (≥ 40% back pain reduction from baseline) could advance to permanent implantation of percutaneous magnetic resonance imaging (MRI)‐compatible octopolar leads and an SCS system (Surescan and Intellis, Medtronic Inc.). Data from subjects who did not achieve a successful trial phase were carried forward toward the primary end point. For permanent implantation, investigators placed permanent percutaneous leads according to the location that rendered the successful trial phase. Physicians followed standard practice of their study site for prophylactic antibiotics and postsurgery analgesics.

Clinical representatives from the study sponsor and Medtronic provided programming support for DTM SCS and traditional SCS, respectively, at the direction of the physician investigators following their own therapy algorithms for optimal pain relief. For traditional SCS stimulation, subjects were programmed according to the labeling/manual. For DTM SCS, subjects were given 3 therapy options to choose from, each consisting of multiple pulsed signals programmed with independent parameters (programs). Each DTM SCS option consisted of 4 electrical signals multiplexed via 4 programs. In general, one program in each DTM SCS option consisted of a 50 Hz signal (200 µs pulse width [PW]) and the other 3 programs of signals at 300 Hz (170 µs PW). Each option delivered the multiplexed signals at different locations in the T8–T11 vertebral region to account for anatomic variabilities of the study subjects. Intensities were set according to a DTM SCS algorithm, starting at a percentage below perception and working them up at regular intervals until reaching therapeutic levels. Subjects adjusted intensity and selected DTM SCS options were based on optimal pain relief.

### Measurements and outcomes

Measurements were 10‐cm visual analog scale (VAS) for 7‐day average back and leg pain scores, Oswestry Disability Questionnaire (ODI), PROMIS Scale version 1.2 Global Health, Patient Global Impression of Change (PGIC), and patient satisfaction with therapy. These, along with clinical descriptions and reports of adverse events (AEs) were collected at baseline, 3‐month, 6‐month, and 12‐month visits. The primary outcome was the comparison of LBP responder rate (percentage of subjects with ≥ 50% decrease in back pain VAS relative to baseline) at the 3‐month visit after device activation for the intention‐to‐treat (ITT) population. Secondary outcomes included comparison of mean change from baseline in LBP VAS at the 3‐month and 6‐month visits, comparison of LBP responder rates at the 6‐month visit, comparison of ODI at the 3‐month visit, and frequency of treatment emergent AEs related to the study. Additional outcomes include comparison of mean change from baseline in LBP VAS and LBP responder rates at 12 months, and comparison of LP responder rates and mean change from baseline in LP VAS at the 3, 6, and 12‐month visits.

### Statistical analysis

Sample size for efficacy was based on noninferiority comparison of the primary end point between treatment groups using a one‐sided Farrington‐Manning binomial test assuming a 10% margin, consistent with analyses from earlier FDA‐approved SCS RCTs,^13^, ^15^ and a 1‐sided 0.05 alpha level, resulting in sample size target of 50 subjects per treatment group (100 total). To account for a combined estimated attrition of 20% for subjects that did not complete the trial phase, and subjects that exited study before the 3‐month primary end point visit, a total of 128 subjects were randomized.

Prespecified analyses for primary and secondary end points were performed based on the statistical plan that was designed a priori.

**FIGURE 1:**
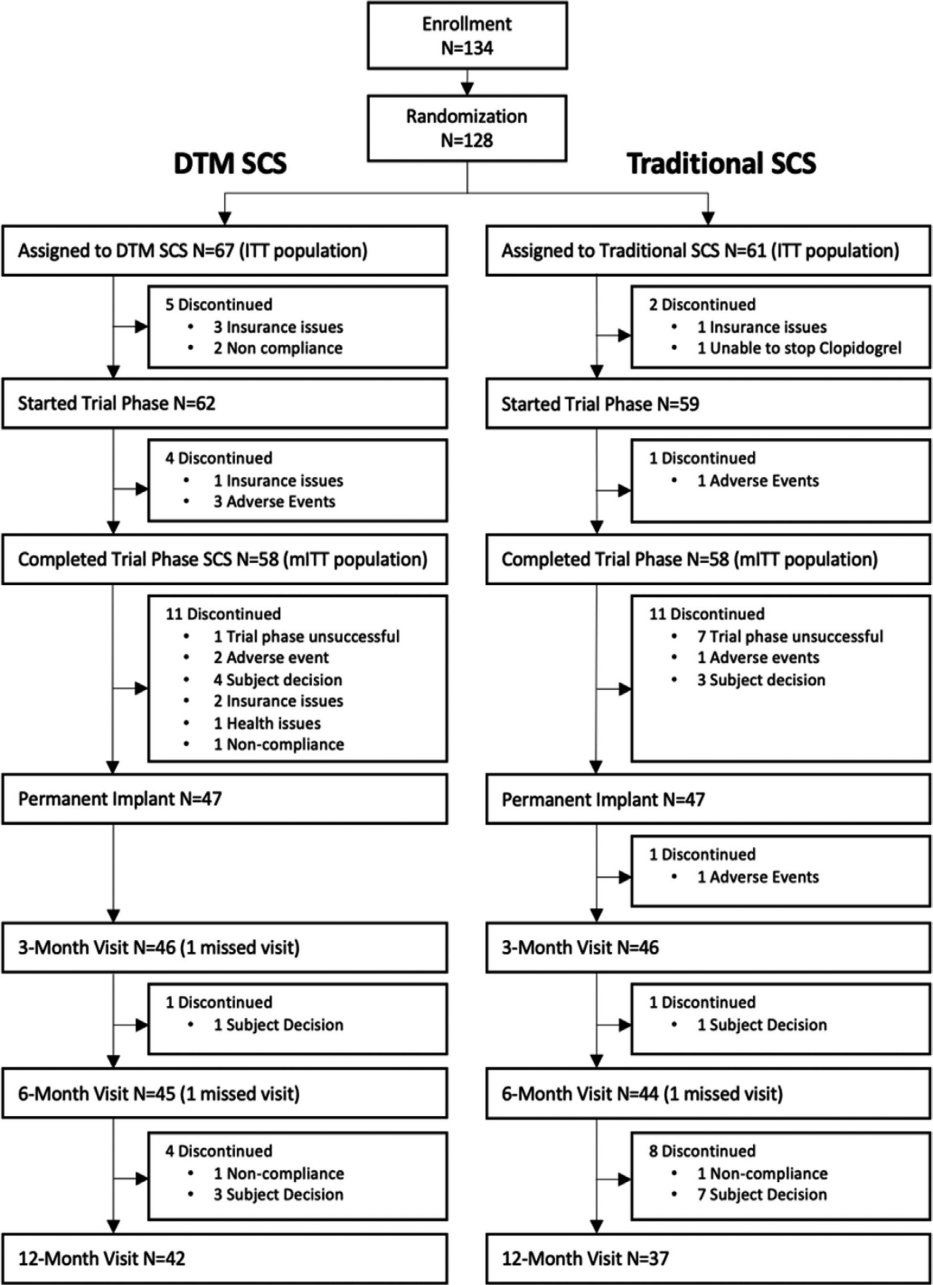
A diagram showing subject disposition throughout the study timeline. DTM, Differential Target Multiplexed; ITT, intention to treat; mITT, modified intention to treat; SCS, spinal cord stimulation

The ITT population, which included all randomized subjects (Figure 1), was used in the analysis of the primary end point. Subjects who did not have a successful trial and those who withdrew for reasons related to lack of pain relief were considered failures toward the primary end point. The impact of other missing data was examined with multiple imputation methods,^21^, ^22^, ^23^, ^24^ as well as sensitivity analyses, consistent with current recommendations on analyses for handling missing data in clinical trials.^24^ The modified ITT (mITT) population, defined as all randomized subjects who completed the trial phase, a completer’s analysis, for subjects with 3‐month results, and a tipping point analysis were used to conduct sensitivity analysis for the primary end point. For the completer’s analysis, subjects with an unsuccessful trial phase and subjects that withdrew before the 3‐month visit due to lack of pain relief were considered nonresponders for the primary end point. The tipping point analysis examined all possible configurations of outcomes (success or failure) for subjects with missing 3‐month results.

If the null‐hypothesis of inferiority were rejected, a 1‐sided Farrington‐Manning binomial test would be performed to assess the superiority of DTM SCS compared to traditional SCS.

Additional secondary outcomes and additional evaluations were assessed using the ITT population with evaluable data. These included LP for subjects with baseline VAS score ≥ 5.0 cm and change of back pain VAS from baseline to 3 months, in which a 1‐sided 2 sample *t*‐test assessed the noninferiority of DTM SCS compared to traditional SCS using a 0.65 cm margin.

Responder rates were further categorized based on the level of pain relief. Recent publications on SCS defined subjects achieving ≥ 80% overall pain relief as “high responders.”^15^, ^25^ We assessed the percentage of subjects who reached ≥ 80% back pain relief and labeled it “profound responders.”

Study‐related AEs (including serious) were reported for the ITT population by treatment group, accounting for the number of events and subjects with event for each event type. Rates were reported as the number of subjects who experienced at least one event during the analysis interval out of the total number of subjects exposed to the trial or permanent devices.

## RESULTS

### Study subjects

Enrollment spanned from June 26, 2018, to August 6, 2019. The study concluded on July 22, 2020. Of the 134 enrolled subjects, 128 were randomized with 67 to the test group and 61 to the control group (ITT population). Of the 94 patients permanently implanted, 92 (46 subjects in each group) completed the 3‐month assessment (Figure 1). In the DTM SCS group, 5 subjects were discontinued after the device implant: one due to noncompliance (cardiac medication) and 4 due to subject decision, which included trying other therapies (1), unrelated health issue (1), and insufficient pain relief (2). There were 10 discontinuations in the traditional SCS group after device implant: 1 due to AE, 1 due to noncompliance (pain medication), and 8 due to subjection decision, which included moving out of state (2), insufficient pain relief (5), and spousal support (1).

Demographic and baseline data are displayed in Table 2. There was a smaller percentage of subjects reporting “lumbar facet‐mediated pain” in the DTM SCS group than in the traditional SCS group (*p* = 0.0030). A post hoc analysis determined that this difference had no meaningful impact on pain outcomes.

**TABLE 2:** Mean demographic information obtained at baseline

Parameter	DTM SCS (*N* = 67)	Traditional SCS (*N* = 61)	*p* value^a^
Gender *n* (%)			
Female	34 (50.7%)	34 (55.7%)	0.60
Male	33 (49.3%)	27 (44.3%)	
Age (years)			
Mean (SD)	61.28 (12.16)	60.66 (11.77)	0.77
Race			
Black or African American	6 (9.0%)	11 (18.0%)	0.19
White	60 (89.5%)	50 (82.0%)	
Other	1 (1.5%)	0 (0.0%)	
Leg pain			
Unilateral	26 (38.8%)	24 (39.3%)	1.00
Bilateral	41 (61.2%)	37 (60.7%)	
Baseline back pain (VAS) (cm)			
Mean (SD)	7.25 (1.49)	7.35 (1.26)	0.67
Baseline leg pain (VAS) (cm)			
Mean (SD)	6.20 (2.58)	6.58 (2.06)	0.36
Pain etiology *n* (%)^b^			
Post‐laminectomy pain syndrome	44 (65.7%)	32 (52.5%)	0.15
Degenerative disc disease	28 (41.8%)	25 (41.0%)	1.00
Lumbar facet‐mediated pain	8 (11.9%)	21 (34.4%)	< 0.01
Spondylolisthesis	4 (6.0%)	4 (6.6%)	1.00
Spondylosis	31 (46.3%)	32 (52.5%)	0.60
Mild/mod spinal stenosis	31 (46.3%)	27 (44.3%)	0.86
Internal disc disruption/annular tear	0/67 (0%)	0/61 (0%)	NA
Radiculopathy	58 (86.6%)	51 (83.6%)	0.80
Sacroiliac dysfunction	7 (10.4%)	9 (14.8%)	0.59
Neuropathic pain	5 (7.5%)	5 (8.2%)	1.00
Other chronic pain	24 (35.8%)	28 (45.9%)	0.28
Approximate number of year(s) since onset of symptoms			
Mean (SD)	12.64 (13.05)	12.89 (11.25)	0.91
Number of spine surgeries			
Mean (SD)	1.49 (1.33)	1.41 (1.13)	0.71

Abbreviations: cm, centimeter; DTM, Differential Target Multiplexed; mod, moderate; NA, not applicable; SCS, spinal cord stimulation; VAS, visual analog scale.

^a^ The *p* values for continuous data were calculated from 2 sample *t*‐test. The *p* values for categorical data were calculated from Fisher’s exact test.

^b^ Percentages do not add to 100% because the subjects reported in more than one category.

About 66% of the enrolled subjects had predominant LBP at baseline, with 72% of the subjects in the DTM SCS group and 61% in the traditional SCS group.

### Trial phase results

One hundred sixteen subjects finished the trial phase, 58 in each group. Mean LBP VAS was 1.34 cm (SD 1.28) at the end of trial in the DTM SCS group, and 2.15 cm (SD 2.10) in the traditional SCS group. Mean change in LBP VAS from baseline to the end‐of‐trial visit were 5.92 cm (SD 1.74) in the DTM SCS group and 5.26 cm (SD 2.25) in the traditional SCS group. The percentage of responders was 98.3% for the test group and 87.9% for the control group.

### Pain relief outcomes

#### Low back pain responder rate

**FIGURE 2:**
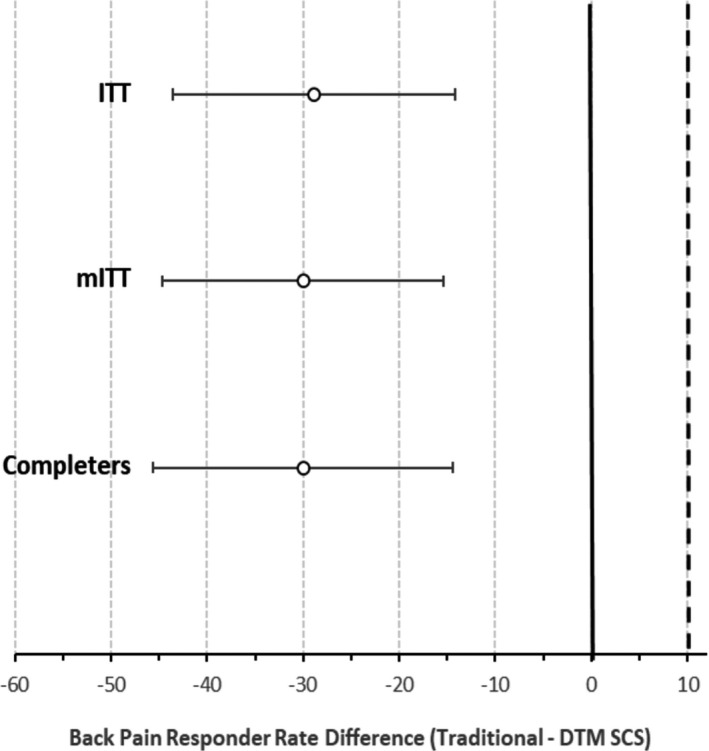
A graph showing treatment differences in back pain responder rates and confidence intervals for analysis populations at the primary end point (3‐month) of the study. The dashed line denotes the noninferiority margin. The bold line denotes the equivalence point. DTM, Differential Target Multiplexed; ITT, intention to treat; mITT, modified intention to treat; SCS, spinal cord stimulation

In the ITT population analysis at the 3‐month visit, the LBP responder rate of 80.1% (90% CI 70.6%–89.7%) in the DTM SCS group (Figure 2) was statistically noninferior to 51.2% (90% CI 40.0%–62.4%) in the traditional SCS group (*p* < 0.0001). A noninferiority test using mITT population yielded similar results: 81.4% for DTM SCS and 51.4% for traditional SCS (*p* < 0.0001). Completer’s analysis yielded responder rates of 80.9% and 50.9% (*p* < 0.0001). Tipping point analyses showed that results were statistically significant in all cases. The worst‐case result, which treated all missing test subjects as failures and all missing control subjects as successes, still produced a *p* value of 0.1042. Thus, the primary end point of the study was met. The LBP responder rate of 80.1% in the DTM SCS group was statistically superior to 51.2% in the traditional SCS group (*p* = 0.0010) using the ITT population analysis at the 3‐month visit. A sensitivity analysis showed that these outcomes were not affected by the use of opioids.

LBP responder rates were sustained throughout the long‐term follow‐up of the study. At the 6‐month visit, these were 73.9% in the DTM SCS group and 50.0% in the traditional SCS group, and at the 12‐month visit, they were 83.7% in the DTM SCS group and 51.1% in the traditional SCS group. This outcome was not affected by the use of additional opioids as well. Only 2 subjects in the control arm and none in the DTM SCS arm were found to have increased their use.

**FIGURE 3:**
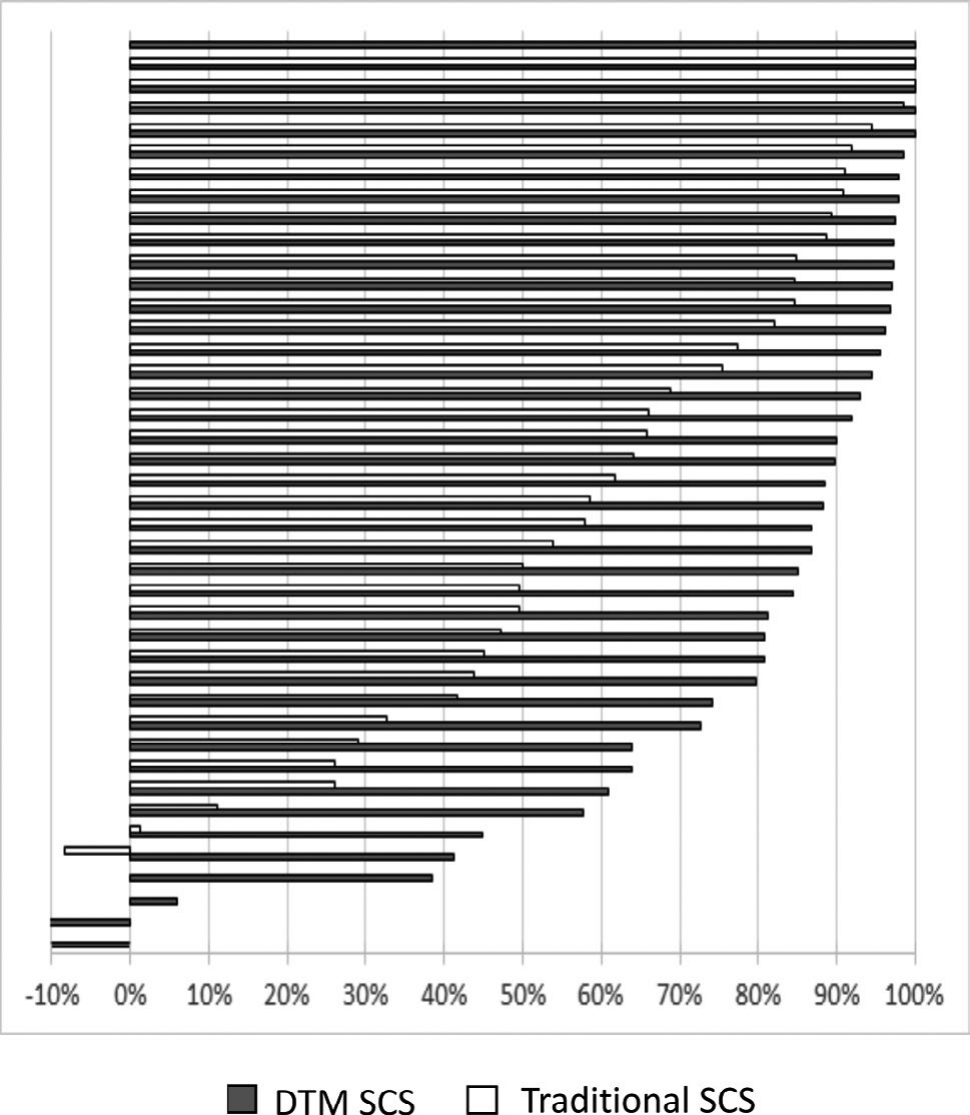
Back pain relief for individual subjects at the 12‐month visit. Sixty‐nine percent of the test subjects (DTM SCS) achieved profound response to back pain. Thirty‐five percent of the control subjects (traditional SCS) achieved profound response to back pain. Profound response is defined as 80% or greater (dashed line) low back pain relief. Analysis included the ITT population with evaluable data at the 12‐month visit. DTM, Differential Target Multiplexed; ITT, intention to treat; SCS, spinal cord stimulation

Furthermore, the profound LBP responder rate (≥ 80% back pain relief) was 63% in the DTM SCS group and 28% in the traditional SCS group at the 3‐month visit, which was sustained at the 12‐month visit, being 69% in the DTM SCS group and 35% in the traditional SCS group (Figure 3).

#### Low back pain scores

**FIGURE 4:**
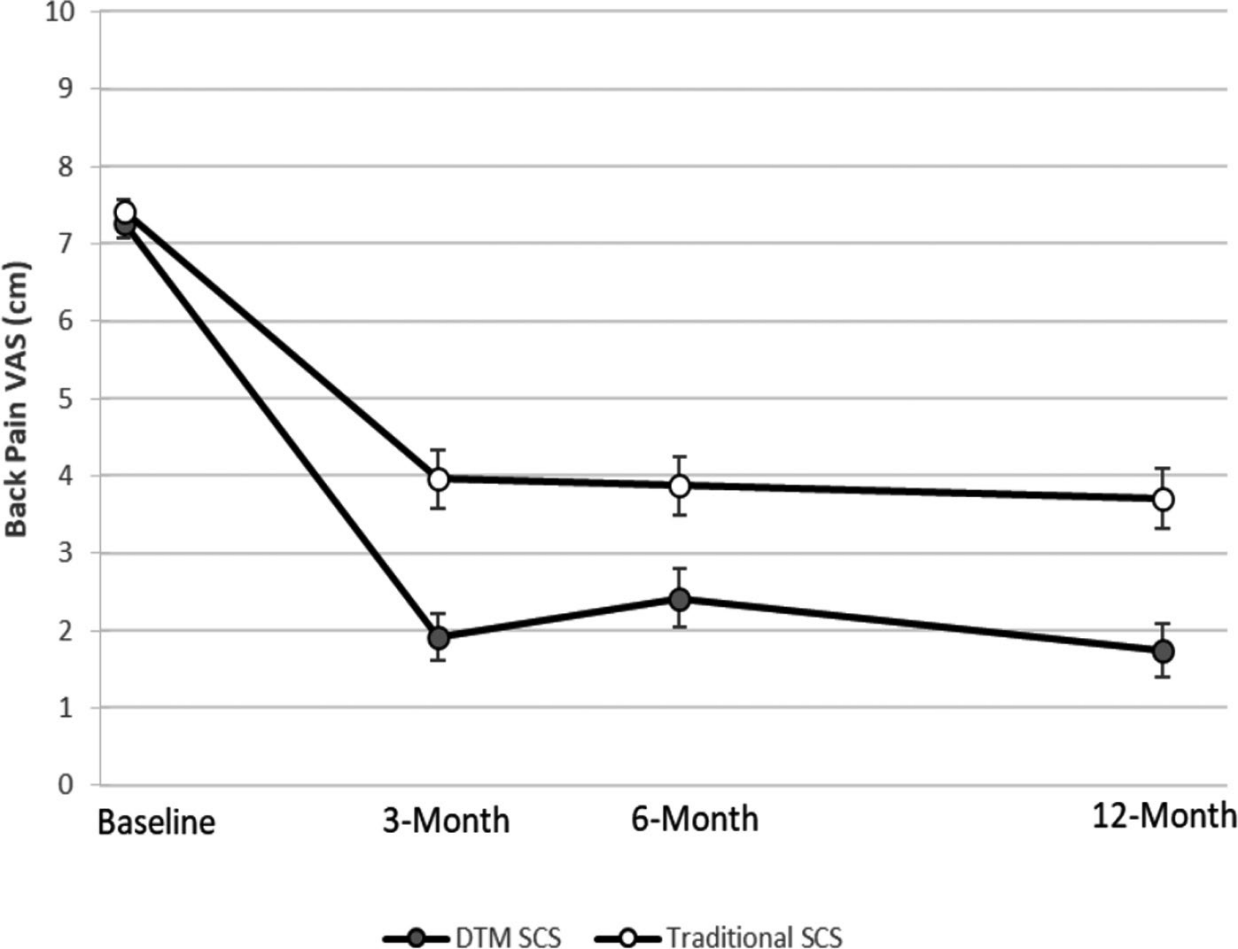
Mean visual analog scale (VAS) scores for longitudinal back pain with standard errors. Values correspond to the analysis with the ITT population with evaluable data at each time point. DTM, Differential Target Multiplexed; ITT, intention to treat; SCS, spinal cord stimulation

Figure 4 shows mean back pain VAS throughout the study duration. The mean reduction of 5.36 cm (SD 2.63) in back pain VAS from baseline to the 3‐month visit with DTM SCS was statistically noninferior to the reduction of 3.37 cm (SD 2.52) with traditional SCS (noninferiority margin = 0.65 cm, *p* < 0.0001). Pain score improvements were sustained throughout the long‐term follow‐up visits. Mean reductions in LBP VAS from baseline to the 6‐month visit were 4.81 cm (SD 2.79) with DTM SCS and 3.47 cm (SD 2.55) with traditional SCS. At the 12‐month visit, these were 5.48 cm (SD 2.69) and 3.62 cm (SD 2.53) with DTM SCS and traditional SCS, respectively.

#### Leg pain scores and responder rates

**FIGURE 5:**
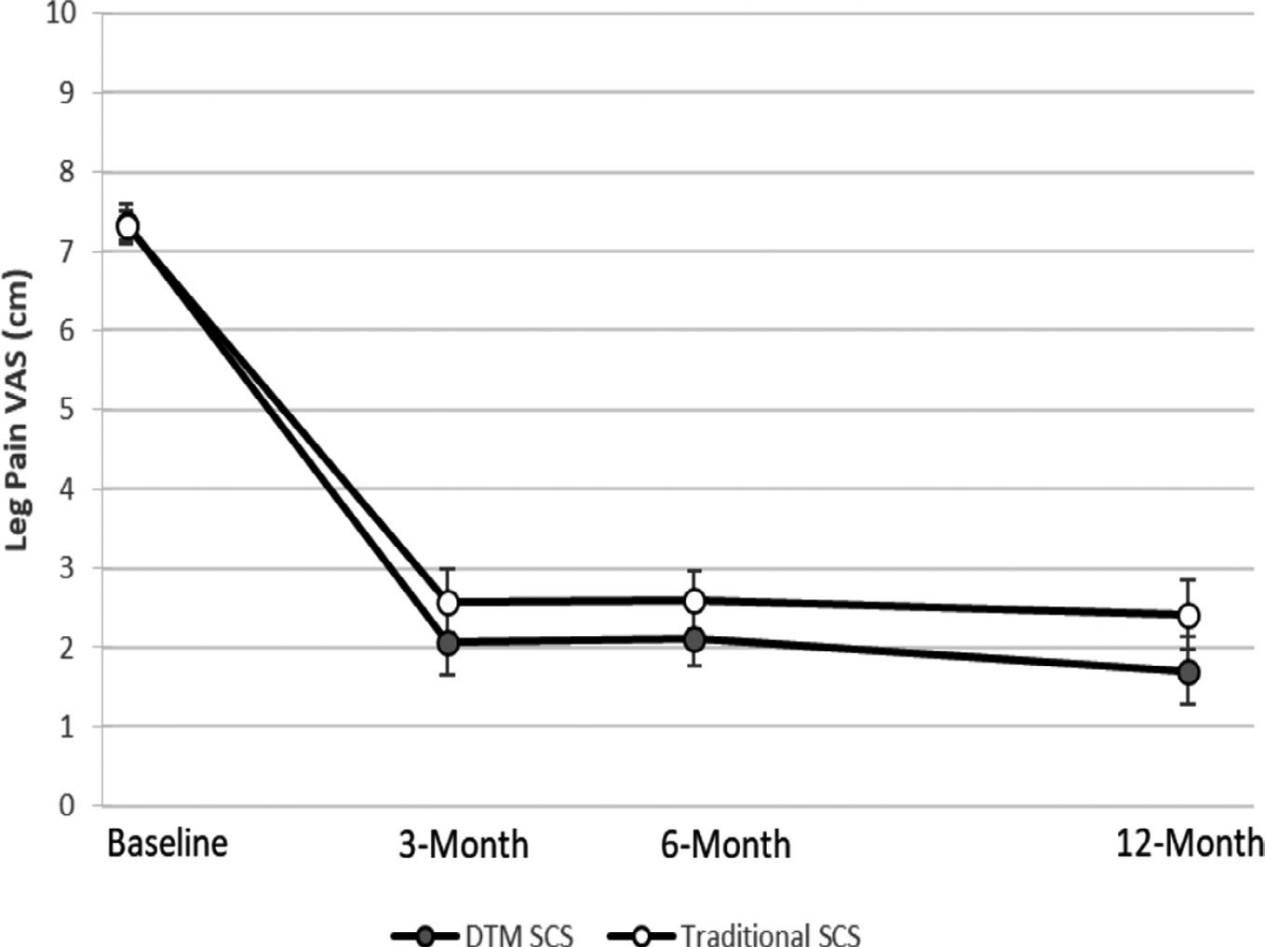
Mean visual analog scale (VAS) scores for longitudinal leg pain with standard errors. Values correspond to the analysis with the ITT population with evaluable data at each time point. DTM, Differential Target Multiplexed; ITT, intention to treat; SCS, spinal cord stimulation

Figure 5 shows mean LP VAS at baseline and along follow‐up visits for subjects that presented baseline LP VAS ≥ 5.0 cm. The mean reductions in LP VAS at the 3‐month visit were 5.29 cm (SD 2.41) with DTM SCS and 4.76 cm (SD 2.52) with traditional SCS. The extent of relief of LP was sustained throughout the follow‐up visits. At the 6‐month visit, mean reductions in LP VAS were 5.21 cm (SD 2.07) with DTM SCS and 4.76 cm (SD 2.26) with traditional SCS. At the 12‐month visit, these were 5.53 cm (SD 2.79) and 4.95 cm (SD 2.38) with DTM SCS and traditional SCS, respectively. LP responder rates (percentage of subjects with ≥ 50% leg pain relief) were 77.1% in the test group and 72.5% in the control group at the 3‐month visit. These were sustained through the 12‐month study visit being 80.0% in the test group and 75.0% in the control arm.

### Safety outcomes

The nature and severity of study‐related AEs were similar between test and control groups (see Table 3). Six percent of subjects in the DTM SCS group and 11.4% of subjects in the traditional SCS group experienced study‐related AEs. There were 24 serious adverse events (SAEs) reported, none of these were deaths. Thirteen of the SAEs were reported for the DTM SCS group, although none were related to the study. Of the 11 reported for the traditional SCS group, 2 were related to the study. One was implant‐site infection resulting in explantation of the device, and the other one was postimplantation exacerbation of LP requiring overnight hospitalization, which subsequently resolved. Among the study‐related AEs (4 from 4 subjects in the test group and 8 from 7 subjects in the control group), the most common included trial lead dislodgement and incisional pain.

**TABLE 3:** Study‐related adverse events

MedDRA preferred term^a^	DTM SCS (*N* = 67)		Traditional SCS (*N* = 61)	
	Number of study‐related AEs	Number (%) of subjects	Number of study‐related AEs	Number (%) of subjects
Total study‐related AEs	4	4 (6.0)	8	7 (11.5)
Abdominal pain	0	0 (0.0)	1	1 (1.6)
Implant site irritation	1	1 (1.5)	0	0 (0.0)
Medical device site pain	0	0 (0.0)	1^c^, ^d^	1 (1.6)
Pain	0	0 (0.0)	1^c^	1 (1.6)
Implant site infection	0	0 (0.0)	1^d^, ^e^	1 (1.6)
Postoperative wound infection	0	0 (0.0)	1	1 (1.6)
Incision site pain	1	1 (1.5)	0	0 (0.0)
Pneumocephalus	0	0 (0.0)	1	1 (1.6)
Procedural complication	0	0 (0.0)	1	1 (1.6)
Lead dislodgement^b^	2^c^	2 (3.0)	0	0 (0.0)
Pruritus	0	0 (0.0)	1	1 (1.6)

Abbreviations: AEs, adverse events; DTM, Differential Target Multiplexed; MedDRA, medical dictionary for regulatory activities; SCS, spinal cord stimulation.

^a^ Events are summarized by MedDRA system organ class (SOC) and preferred term (PT). The sum of the subjects need not sum to total as subjects may experience more than one type of event.

^b^ Both were trial phase events.

^c^ Events were reported during the trialing period and the subjects who discontinued.

^d^ Serious adverse event.

^e^ Led to system explant.

### Other outcomes

ODI was used to measure the functional disability level of subjects. At baseline, 27% of subjects in the DTM SCS group and 25% in the traditional SCS group had minimal or moderate disability. At 12 months, these percentages increased to 76% in the DTM SCS group compared to 62% in the traditional SCS group (Table 4).

**TABLE 4:** Oswestry Disability Index (ODI) categories

	DTM SCS				Traditional SCS			
	Baseline (%)	Month 3 (%)	Month 6 (%)	Month 12 (%)	Baseline (%)	Month 3 (%)	Month 6 (%)	Month 12 (%)
Minimal	0.0	34.8	28.9	31.0	0	28.3	27.3	32.4
Moderate	26.9	32.6	53.3	45.2	24.6	39.1	36.4	29.7
Severe	56.7	26.1	13.3	21.4	55.7	23.9	31.8	37.8
Crippled/bedbound	16.4	6.5	4.4	2.4	19.7	8.7	4.5	0.0

Abbreviations: DTM, Differential Target Multiplexed; SCS, spinal cord stimulation

Subject’s perception of quality of life was evaluated using the PROMIS scale. At baseline, 39% of subjects in the DTM SCS group were excellent, very good, good, or fair, and 36% of subjects in the traditional SCS group. At 12 months, these percentages increased to 88% for the DTM SCS group, in comparison to 76% for the traditional SCS group (Table 5).

**TABLE 5:** PROMIS global health categories

	DTM SCS				Traditional SCS			
	Baseline (%)	Month 3 (%)	Month 6 (%)	Month 12 (%)	Baseline (%)	Month 3 (%)	Month 6 (%)	Month 12 (%)
Excellent/very good	0.0	13.3	15.6	7.1	0.0	10.9	18.2	18.9
Good	4.5	33.3	31.1	45.2	9.8	37.0	25.0	27.0
Fair	34.3	26.7	37.8	35.7	26.2	15.2	25.0	29.7
Poor	61.2	26.7	15.6	11.9	63.9	37.0	31.8	24.3

Abbreviations: DTM, Differential Target Multiplexed; SCS, spinal cord stimulation

Satisfaction and PGIC assessments were both high in the DTM SCS and traditional SCS groups. The percentages of subjects who rated “very satisfied” were 41.3% for the DTM SCS group and 39.1% for the traditional SCS group at the 3‐month visit; at the 12‐month visit, the percentages increased to 61.9% for the DTM SCS group and 45.9% for the traditional SCS group. In PGIC assessments, the percentages of subjects reporting “a great deal better” was 30.4% for the DTM SCS group and 21.7% for the traditional SCS group at the 3‐month visit and were increased to 42.9% for the DTM SCS group and 29.7% for the traditional SCS group at the 12‐month visit.

## DISCUSSION

This study demonstrated that DTM SCS provided superior LBP relief compared with traditional SCS programming in patients with intractable LBP and LP at the 3‐month visit in the ITT population. The study demonstrated sustained benefits with high efficacy in LBP out to 12 months. Responder rates for LBP at the 3‐month visit (80.1%) and the 12‐month visit (83.7%) were among the highest compared with other RCTs in the field, including newer technologies.^13^, ^15^ Mean back pain VAS was lowered to 1.91 cm at the 3‐month visit and sustained to 1.74 cm at the 12‐month visit. The high responder rate and mean back pain VAS below 2 cm are notable given the high chronicity of pain (mean of 12.64 years) and level of baseline pain (mean 7.25 cm) in the DTM SCS group. Additionally, the patient population is older than in previously reported RCTs,^9^, ^13^, ^14^, ^15^ a population that is considered to be challenging.

LP scores improved in both groups and were not statistically different at 3 or 12 months, although mean VAS tended to be lower with DTM SCS. At 3 months, the mean LP VAS was low with both DTM SCS (2.07 cm) and traditional SCS (2.58 cm). This improvement was sustained through 12 months (1.71 and 2.42 cm, respectively). This speaks to the efficacy of traditional SCS in relieving neuropathic leg pain and is consistent with previous findings.^7^, ^8^ As such, there may be an advantage in being able to deliver both stimulation approaches with a single implanted impedance plethysmogram (IPG).

Consistent with pain relief, DTM SCS improved the quality of life and reduced disability. The percentage of subjects who were excellent, very good, good, or fair in terms of quality of life expanded from 39% at baseline to 88% at the 12‐month visit. Likewise, the percentage of subjects who were in minimal or moderate disability greatly improved from 27% at baseline to 76% at the 12‐month visit.

An mITT population served as an evaluation group that excluded subjects who were randomized (ITT population) but did not start the trial phase. This population excluded subjects who could not get reimbursement approvals, who were discontinued by the investigator due to noncompliance, and a subject who could not continue due to anticoagulant use. Regardless, the primary outcome of noninferiority was the same when using an analysis based on either population (ITT or mITT).

Although attrition for the DTM SCS group was within the expected range, it should be noted that the traditional SCS group experienced a higher rate of loss to follow‐up for the 12‐month visit. Notably, only one subject discontinued between device implant and the 3‐month visits, and one subject discontinued between the 3‐month and the 6‐month visits. Eight more discontinued between the 6 and the 12‐month visits. Half of traditional SCS subjects (5/10) who discontinued after device implant were not responding to the therapy.

Although DTM SCS demonstrated superior LBP efficacy compared to traditional SCS, it is worth noting that traditional SCS performed well. At the 12‐month visit, traditional SCS achieved 51% responder rate. This is consistent with the 51% responder rate achieved by traditional SCS at the 12‐month visit in the HF10 RCT^13^ and the 58% responder rate at the 12‐month visit in the EVOKE RCT.^15^ In addition, it is notable that 35% of the control subjects were profound responders in this study, similar to that reported (37%) in a recent study for overall pain,^15^ making traditional SCS a useful option.

The results are remarkable considering that a particularly difficult treatment population was enrolled. Axial LBP is known to be particularly difficult to treat because of its mixed nociceptive and neuropathic nature.^5^ The first SCS RCT for failed back surgery syndrome excluded patients with predominant LBP.^7^ Since then, attempts have been made to treat predominant LBP with new technologies and approaches. A recent landmark SCS RCT using new technology reported enrolling about 55% of subjects with predominant LBP at baseline (56% in the test arm and 53% in control arm).^13^ In this study, 72% of the DTM SCS subjects had predominant LBP.

The incidence of device‐related AEs and SAEs were consistent with other SCS studies.^26^, ^27^, ^28^ There were no SAEs related to DTM SCS. Of note, lead migrations occurred during the trial phase. The one SAE, infection at the implant site, reported in the control group is consistent with standard SCS practice. The other SAE in the control group, exacerbation of LP during implantation, resolved after one night of hospitalization.

Preclinical research utilizing rodent and ovine models of neuropathic pain showed that DTM‐based programs (DTMPs) provided better relief of pain‐like behavior than traditional‐based programs, which utilized one single electrical signal.^29^, ^30^ Analysis of neuron‐specific and glia‐specific gene expression in a rodent model demonstrated that treatment with DTMP modulated gene expression of such cell‐specific transcriptomes toward levels found in naïve animals.^18^ This supported the hypothesis that DTM SCS provides analgesia by balancing pain‐related biological processes, such as neuroinflammation and ion transport, which involve neuron‐glial interaction that had been affected by the establishment of neuropathic pain.^17^

### Limitations

Due to the nature of the programs, it was not feasible to blind subjects, implanting physicians, or clinical site personnel to the group assignments. Subjects assigned to the traditional SCS were programmed to have optimal pain relief while adjusting intensities to maintain comfortable stimulation, whereas those in the DTM arm were able to titrate intensities below the perception threshold according to the programming algorithm in order to get optimal pain relief. Efforts were made to reduce bias where possible. Each SCS group was programmed under the direction of physicians with support of different clinical representatives to provide analgesia for each group; sponsor’s representatives supporting DTM SCS and device manufacturer’s representatives supporting traditional SCS. When programming support of both treatments is provided by representatives of a single organization, it is plausible that they may be biased to provide better care for the test arm than the control arm. Additionally, assessment of the treatment performance was done by subjects and not by the site personnel.

Salvaging patients who have failed to respond to SCS is an area gaining attention as new SCS approaches are introduced. This was not assessed in this study because the focus was on understanding the effects of DTM SCS in patients naïve to SCS. A key exclusion in this study was having an active implanted device, such as an SCS system, whereas control subjects who failed the trial phase were not given an option to crossover. A separate RCT is needed to further study this subset of patients. A study with a crossover design including an appropriate washout period may be helpful in further understanding comparison of patient satisfaction outcomes.

## CONCLUSION

Differential target multiplexed SCS was statistically noninferior (*p* < 0.0001) and superior (*p* = 0.0010) to traditional SCS in the ITT population. Significantly greater reduction in mean LBP VAS relative to baseline was observed to 12 months, demonstrating that DTM SCS provided robust and positive benefits that were sustained over time. Both treatment groups experienced meaningful LP relief as well as improvements from baseline in measures of disability.^31^ These results were consistent over the course of 12‐months of follow‐up visits. The frequency, type, seriousness, and severity of study‐related AEs were similar and demonstrated an acceptable risk profile in both treatment groups. The superior benefits of the DTM SCS programming offers clinicians and patients a highly effective option for the treatment of intractable chronic back pain.

## CONFLICT OF INTERESTS

J.C. reports consulting fees from PillNurse; honoraria or payment for lectures/speaker bureau/educational events from Abbot, Biotronik, Boston Scientific, Medtronic, and Saluda; medical advisory board membership in Mainstay Corneloc, and PillNurse; treasurer and executive board member of ASPN, president of AZSIPP; and stock/stock options in Cornerloc and Mainstay. M.F. reports research grants to institution from Abbott, Biotronik, Boston Scientific, Medtronic, Nalu Medical, SGX Medical, and Thermaquil; consulting fees from Abbott, Biotronik, Medtronic, and Nevro; faculty appointment with Abbott and Medtronic; director‐at‐large of NANS; and stock/stock options at Celeri Health, and Thermaquil. R.J. reports honoraria or payment for speaker bureau from Medtronic. P.K. reports honoraria or payment for lectures/speaker bureau/educational events from Biotronik and Medtronic. V.M. reports consulting fees from Aurora Spine and Medtronic; and stock/stock options from Aurora Spine. D.P. reports consulting fees from Avanos, Boston Scientific, Esteve, Heron, Medtronic, and Nevro; and other financial or nonfinancial interests from Abbott, Avanos, Medtronic, Nevro, and Stimgenics. M.S. reports being secretary of ASIPP. R.V. reports research grant from Medtronic, consulting fees from Stimgenics (CEO) and SGX Medical (CEO); honoraria or payment for speaker bureau from Medtronic; patents granted/pending assigned to Medtronic; medical advisory board membership in Medtronic; past director‐at‐large of NANS; stock/stock options at SGX Medical. R.B., A.C., H.C., J.F., C.M., and B.S. report no competing interests.

## AUTHOR CONTRIBUTIONS

All authors were responsible for the content and have read, revised and approved the final draft of the manuscript submitted to the journal.

## Data Availability

The data was available for review by all the contributing authors.
